# Overexpression of the MexXY Multidrug Efflux System Correlates with Deficient Pyoverdine Production in *Pseudomonas aeruginosa*

**DOI:** 10.3390/antibiotics10060658

**Published:** 2021-05-31

**Authors:** Kei Ikarashi, Ryo Kutsuna, Junko Tomida, Yoshiaki Kawamura, Yuji Morita

**Affiliations:** 1 Department of Infection Control Science, Meiji Pharmaceutical University, Tokyo 204-8588, Japan; m196202@std.my-pharm.ac.jp; 2 Department of Microbiology, School of Pharmacy, Aichi Gakuin University, Aichi 464-8650, Japan; kutsuna@dpc.agu.ac.jp (R.K.); jtomida@dpc.agu.ac.jp (J.T.); kawamura@dpc.agu.ac.jp (Y.K.)

**Keywords:** *Pseudomonas aeruginosa*, MexXY multidrug efflux system, pyoverdine production

## Abstract

Multidrug-resistant *Pseudomonas aeruginosa* poses a serious problem due to hospital- and healthcare-associated infections. A major drug resistance mechanism of *P. aeruginosa* involves active efflux via resistance nodulation cell division (RND)-type multidrug efflux pumps of which MexXY is increasingly recognized as a primary determinant of aminoglycoside resistance in *P. aeruginosa*. MexXY overexpression is often observed in drug-resistant *P. aeruginosa* clinical isolates. MexXY deficiency increased pyoverdine production in all four *P. aeruginosa* strains we tested. MexXY-overproducing multidrug-resistant *P. aeruginosa* PA7 exhibited the greatest effect among the strains. Complementation with a MexXY-expressing plasmid restored low-level pyoverdine production in a MexXY-deficient *P. aeruginosa* mutant from PA7, indicating that MexXY expression decreases pyoverdine production. Because *P. aeruginosa* produces pyoverdine to acquire iron, MexXY-deficient mutants might be more susceptible to iron deficiency than MexXY-producing strains or might require extra iron. High-risk clones of multidrug-resistant *P. aeruginosa* reportedly tend to be MexXY overproducers but defective pyoverdine producers. This study suggests that *P. aeruginosa* reduces production of a virulence factor after acquiring a drug resistance factor.

## 1. Introduction

*Pseudomonas aeruginosa* is a known opportunistic pathogen and a major threat in hospital and healthcare-associated environments [1]. Infections caused by *P. aeruginosa* are often difficult to treat; inappropriate chemotherapy readily selects multidrug-resistant *P. aeruginosa* strains against which very few agents are effective [2,3]. A major factor in the prominence of *P. aeruginosa* as a pathogen is its intrinsic resistance to various antibacterial agents [2,3]. One of most important chromosomally encoded antimicrobial resistance factors in *P. aeruginosa* is resistance nodulation cell-division (RND)-type multidrug efflux pumps [2,4]. Among these pumps, the MexXY system is the only significant determinant of efflux-mediated aminoglycoside resistance in *P. aeruginosa* [5]. In addition to aminoglycosides, MexXY mediates resistance to other clinically relevant drugs such as cefepime, ciprofloxacin, tigecycline, azithromycin, and colistin [5,6].

Worldwide epidemic outbreaks of infection with highly drug-resistant *P. aeruginosa* are often associated with various so-called international high-risk clones [7], many of which harbor chromosomal mutations that promote drug resistance mechanisms, such as MexXY overproduction [8]. These clones also often produce lower amounts of pyoverdine in vitro, which has been proposed as a potential biomarker [9]. Pyoverdines that facilitate acute infections by pseudomonads include fluorescent siderophores, which specifically chelate Fe^3+^ with high affinity [10]. However, the types of gene mutations that contribute to defective pyoverdine production in highly multidrug-resistant *P. aeruginosa* clinical isolates remain unknown, as no major differences in pyoverdine gene clusters have been identified [11]. In this study, we conducted a detailed examination of the effect of MexXY on the production of pyoverdine in *P. aeruginosa*.

## 2. Results

**Figure 1:**
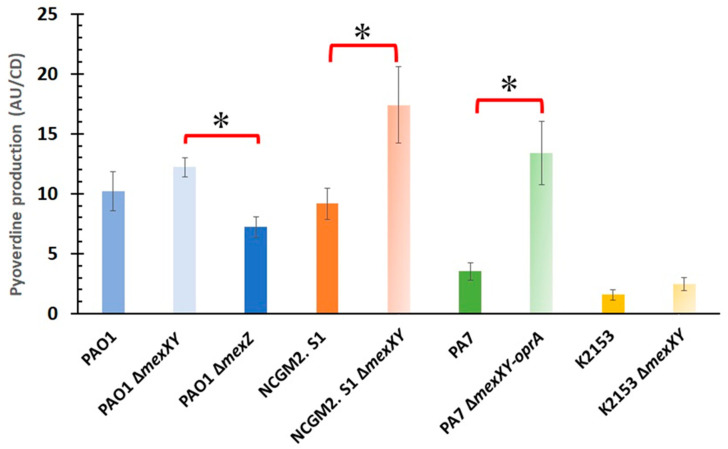
The MexXY multidrug efflux system decreased pyoverdine production in *P. aeruginosa*. Pyoverdine production was calculated by dividing the fluorescence value (arbitrary units, AU) by cell density (CD). The error range shown in the bar graph is the standard error (*n* = 5). * Indicates *p* < 0.05.

During antimicrobial susceptibility tests of broth microdilution MIC methods (e.g., [12]) we found that strain PA7 Δ*mexXY-oprA* mutant were more yellow-green in color than those of the PA7 parent strain, which are highly multidrug resistant [13]. Therefore, we quantitatively examined pyoverdine production by four *P. aeruginosa* strains in comparison with the corresponding *mexXY*-deficient mutants (Figure 1). Of note, in our pyoverdine production assay system, fluorescence emission from PAO1 Δ*pvdA* [14] was almost negligible compared with the parent strain, PAO1 (data not shown). PA7 and K2153, a pan-aminoglycoside-resistant strain [15], exhibited markedly defective pyoverdine activity compared with PAO1, a drug-sensitive strain [15], whereas NCGM2. S1, a highly multidrug-resistant strain [16], exhibited slightly but reproducibly lower pyoverdine production than strain PAO1 (Figure 1). Of note, both PA7 and K2153 are *mexXY* overproducers, whereas PAO1 and NCGM2. S1 are not [12,15].

In all four strains examined, pyoverdine production increased due to MexXY deficiency (Figure 1). In particular, deficiency had the greatest impact on pyoverdine production in PA7 (3.8-fold increase), whereas MexXY deficiency was associated with a 1.2-fold increase in PAO1, a 1.9-fold increase in NCGM. 2 S1, and a 1.6-fold increase in K2153. In addition, deletion of *mexZ*, a local repressor gene of *mexXY* [17,18], resulted in a 1.7-fold decrease in pyoverdine production in strain PAO1 (Figure 1). Statistically significant differences in pyoverdine production were observed between PAO1 Δ*mexXY* and PAO1 Δ*mexZ* (*p*-value: 0.024 [<0.05]), between NCGM2. S1 and NCGM2. S1 Δ*mexXY* (*p*-value: 0.032 [<0.05]), and between PA7 and PA7 Δ*mexXY-oprA* (*p*-value: 0.008 [<0.05]). No significant difference was observed, however, between K2153 and K2153 Δ*mexXY* (*p*-value: 0.55 [>0.05]).

**Figure 2:**
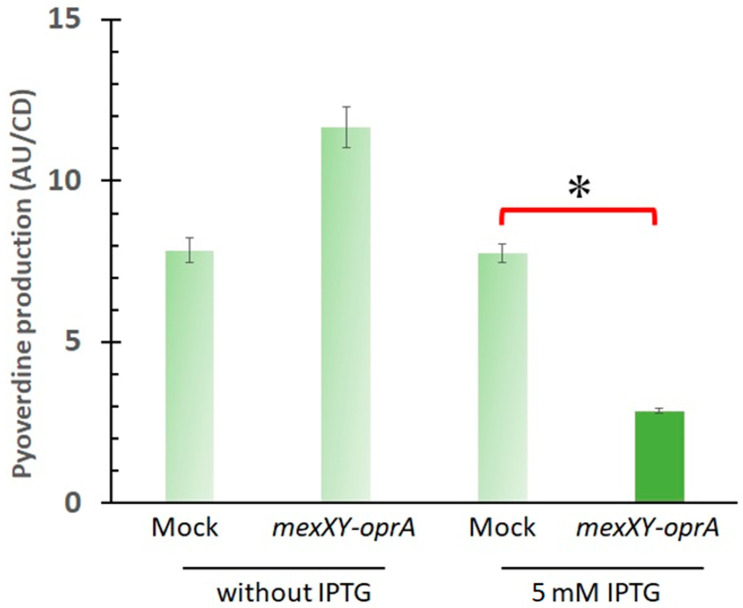
IPTG-induced *mexXY-oprA* expression decreased pyoverdine production in *P. aeruginosa*. Pyoverdine production was calculated by dividing the fluorescence value (arbitrary units, AU) by cell density (CD). Mock and *mexXY-oprA* on the horizontal axis indicate two complemented strains: PA7 Δ*mexXY-oprA attB*::pYM101 and PA7 Δ*mexXY-oprA attB*::pYM101-*mexXY-oprA*, respectively (Table 1). The error ranges shown in the bar graph are the standard error (*n* = 5). * Indicates *p* < 0.05. Cultures were supplemented with 5 mM IPTG by addition to *Pseudomonas* agar F to drive the T7(A1/04/03) promoter expression system fully [12,19].

To confirm that the phenotypic changes due to *mexXY* deletion were actually due to loss of function of MexXY, we examined pyoverdine production by the complemented strain in which a *mexXY-oprA* expression plasmid was introduced into PA7 Δ*mexXY-oprA*, in comparison with the negative control strain (Figure 2). Upon addition of isopropyl β-D-1-thiogalactopyranoside (IPTG) to induce plasmid-driven *mexXY-oprA* expression, the *mexXY-oprA-*expressing strain produced 2.7-fold more pyoverdine than the negative control strain (*p* value: 0.008 [<0.05]). In contrast, without IPTG, no significant difference in pyoverdine production was observed between the strains (*p* value: 0.30 [>0.05]).

## 3. Discussion

The results of this study suggest that the MexXY multidrug efflux system decreases pyoverdine production in *P. aeruginosa*, indicating that MexXY-producing *P. aeruginosa* cells require less iron than MexXY-deficient cells, because *P. aeruginosa* requires pyoverdine for survival when iron concentrations become low [20]. The promotion of *mexXY* expression under conditions of oxidative stress is similar to the case of *P. aeruginosa* infection of chronically inflamed lungs of cystic fibrosis (CF) patients [21], which also induces siderophore biosynthesis genes [22], possibly due to oxidative inactivation of the Fur-Fe^2+^ complex [23]. A further study is thus necessary to elucidate the molecular mechanisms in more detail. A whole genome analyses of the studied *P. aeruginosa* strains can be one approach to use which might shed light and allow to get a broad picture.

Another possibility is that pyoverdine could be a substrate of MexXY, rather than MexXY decreasing pyoverdine production. The approach used for examining pyoverdine production does not rule out this possibility, since cells are taken from solid media, suspended in solution and then pyoverdine in the solution measured fluorometrically. Pyoverdine exported by cells on the solid media would presumably diffuse into the agar and thus might not be associated with cells collected for the assay although preliminary results from liquid culture supports the agar results in this study (Figure S1). If MexXY promotes pyoverdine efflux, *mexXY* deletion mutants could have higher cytosolic pyoverdine concentrations than the parental strains and carry more pyoverdine into the assay. The results would be more convincing if the cells were grown in liquid media and the spent media was assayed for pyoverdine concentration. It is also noteworthy that the buffer used for the pyoverdine assay contain the only contaminated iron. The absorbance spectra of free pyoverdine and iron-loaded pyoverdine are different. The maximum absorbance of iron-loaded pyoverdine is 400 nm (as used for excitation in the assay), but the maximum absorbance for unloaded pyoverdine is lower and 400 nm would only hit the shoulder. Therefore, having the only contaminated iron in the buffer might also impact the results of the assay.

Defective pyoverdine production is a biomarker of high epidemic risk *P. aeruginosa* clones [9], and multidrug resistant *P. aeruginosa* clinical isolates [5], including high epidemic risk clones [8], often overexpress *mexXY*. Decreased pyoverdine production could be, at least in part, due to *mexXY* overexpression in *P. aeruginosa* clinical isolates. We cannot rule out the possibility that other factors also contribute to defective pyoverdine production in *P. aeruginosa* clinical isolates. For example, *mexXY* expression does not appear to be the primary reason for defective pyoverdine production in *P. aeruginosa* K2153, a pan-aminoglycoside-resistant clinical isolate obtained from a CF patient (Figure 1). This study provides experimental evidence that upregulated expression of a drug-resistance factor leads to decreased production of a virulence factor in *P. aeruginosa*.

## 4. Materials and Methods

### 4.1. Pseudomonas aeruginosa Strains and Growth Conditions

*Pseudomonas aeruginosa* strains used in this study are listed in Table 1. Bacteria were grown in an Air-Jacketed Incubator IC802 (Yamato Scientific Co., Ltd., Tokyo, Japan) at 37 °C under aerobic conditions, as previously described [12]. Unless otherwise indicated, bacteria were cultured using lysogeny broth, Lennox (LB)-agar prepared fresh from 1.0% Bacto^TM^ Tryptone (Becton, Dickinson and Company, Franklin Lakes, NJ, USA), 0.5% Bacto^TM^ yeast extract (Becton, Dickinson and Company, Franklin Lakes, NJ, USA), and 0.5% NaCl (FUJIFILM Wako Pure Chemical Corp., Osaka, Japan). *Pseudomonas* agar F was prepared from 2.0% Bacto^TM^ proteose peptone no. 3 (Becton, Dickinson and Company, Franklin Lakes, NJ, USA), 1.0% Bacto^TM^ Casitone (Becton, Dickinson and Company, Franklin Lakes, NJ, USA), 1.0% glycerol (Nacalai Tesque Inc., Kyoto, Japan), 0.15% K_2_HPO_4_ (FUJIFILM Wako Pure Chemical Corp., Osaka, Japan), 0.073% MgSO_4_⋅7H_2_O (FUJIFILM Wako Pure Chemical Corp.), and 1.5% agar (FUJIFILM Wako Pure Chemical Corp.), and used as a solid medium in STAR SDish9015 ver.2 petri dishes (Rikaken Co., Ltd., Nagoya, Japan) for assays of pyoverdine production. Bacterial growth was quantified by measuring the optical density at 600 nm (OD_600_) using a WPA CO8000 Cell Density Meter (Biochrom Ltd., Cambridge, UK).

### 4.2. Assay of Pyoverdine Production

Pyoverdine production was assayed according to a previously reported method [9]. Bacteria grown overnight at 37 °C were suspended in 0.85% sterilized NaCl using a sterile wooden-axis cotton swab (Eiken Chemical Co., Ltd., Tokyo, Japan) and diluted to a final OD_600_ of 0.001. Next, 100 μL of the diluted culture was plated uniformly onto *Pseudomonas* F agar using a spreading stick and incubated at 37 °C for 14 h. Colonies of *P. aeruginosa* grown on the agar were suspended in 0.85% sterilized NaCl using a sterile wooden-axis cotton swab. After the cell density (OD_600_) (A) was measured, the suspension was centrifuged at 23 °C and 4000× *g* for 10 min. Each of three 200-μL aliquots from the supernatant was transferred into each of three wells of a 96-well plate (Costar^®^ no lid black, flat bottom, non-treated polystyrene (Corning Inc., Corning, NY, USA). The fluorescence emission for each well was measured (B) at an excitation wavelength of 400 nm and an emission wavelength of 460 nm using a SpectraMax iD3 multi-mode microplate reader (Molecular Devices, LLC, San Jose, CA, USA). Pyoverdine production was calculated by dividing (B) by (A). Each experiment was performed independently at least five times. When necessary, *Pseudomonas* agar F was supplemented with 5 mM IPTG (FUJIFILM Wako Pure Chemical Corp.).

Statistical analyses were performed using the R software version 4.0.4 (https://www.r-project.org/) with the Wilcoxon rank test or Steel–Dwass test. A *p*-value of <0.05 was judged as indicating statistical significance.

## Figures and Tables

**Table 1:** *Pseudomonas aeruginosa* strains used in this study.

Lab Stock	Strain (=Co-Identical Strain) *	Reference
IMPU 1	PAO1 (=K767 or PAGU 974)	[15]
IMPU 2	NCGM2. S1 (=PAGU 1606)	[16]
IMPU 9	PAO1 ΔmexXY (=K1525, PAGU 975)	[15]
IMPU 10	NCGM2. S1 ΔmexXY (=PAGUg1659)	[12]
IMPU 17	PAO1 ΔmexZ (=K2415, PAGUg1659)	[17]
IMPU 21	PA7 (=PAGU 1498)	[13]
IMPU 29	K2153 (=PAGU 1741)	[15]
IMPU 44	PA7 ΔmexXY-oprA (=PAGUg1565)	[12]
IMPU 45	K2153 ΔmexXY (=PAGUg1857)	[15]
IMPU 53	PA7 ΔmexXY-oprA attB::pYM101 (=PAGUg1632)	[12]
IMPU 54	PA7 ΔmexXY-oprA attB::pYM101-mexXY-oprA (=PAGUg1633)	[12]
IMPU 61	PAO1	[14]
IMPU 62	PAO1ΔpvdA	[14]

* Co-identical strain is defined as a strain when stocked in the previous lab(s).

## Supplementary Materials

The following are available online at https://www.mdpi.com/article/10.3390/antibiotics10060658/s1, Figure S1. The MexXY multidrug efflux system decreased pyoverdine production from liquid culture in *P. aeruginosa.*
