# Supplementary material for: Overexpression of the MexXY Multidrug Efflux System Correlates with Deficient Pyoverdine Production in Pseudomonas aeruginosa
Source: Antibiotics (Basel). 2021 May 31;10(6):658. doi: 10.3390/antibiotics10060658 (PMC8226967; doi:10.3390/antibiotics10060658)
Supplement: Supplementary file 1 [file antibiotics-10-00658-s001.zip › antibiotics-1208264-supplementary.pdf]

Assay of pyoverdine production from liquid culture

Pyoverdine production was assayed according to a previously reported method [9]. Bacteria grown overnight at 37 °C were suspended in 0.85% sterilized NaCl using a sterile wooden-axis cotton swab (Eiken Chemical Co., Ltd, Tokyo, Japan) and diluted to a final OD_{600} of 0.1. 30 μL of the diluted culture was inoculatedinto3 mL of Pseudomonas F broth and then shaken vigorously at 37 °C for 7hto reach early to middle logarithmic phase. After the cell density (OD_{600}) (A) was measured, the culture was centrifuged at 23 °C and 4000*g* for 10 min. Each of three 200-μL aliquots from the supernatant was transferred into each of three wells of a 96-well plate (Costar® no lid black, flat bottom, non-treatedpoly-styrene (Corning Inc., Corning, NY, USA). The fluorescence emission for each well was measured (B) at an excitation wavelength of 400 nm and an emission wavelength of 460 nm using a SpectraMax iD3 multi-mode microplate reader (Molecular Devices, LLC, San Jose, CA, USA). Pyoverdine production was calculated by dividing (B) by (A). Each experiment was performed independently at least five times. When necessary, Pseudomonas agar F was supplemented with 5 mM IPTG (FUJIFILM Wako Pure Chemical Corp.). Statistical analyses were performed using R software version 4.0.4 (https://www.r-project.org/) with the Wilcoxon rank test or Steel-Dwass test. A *p*-value of <0.05 was judged as indicating statistical significance.

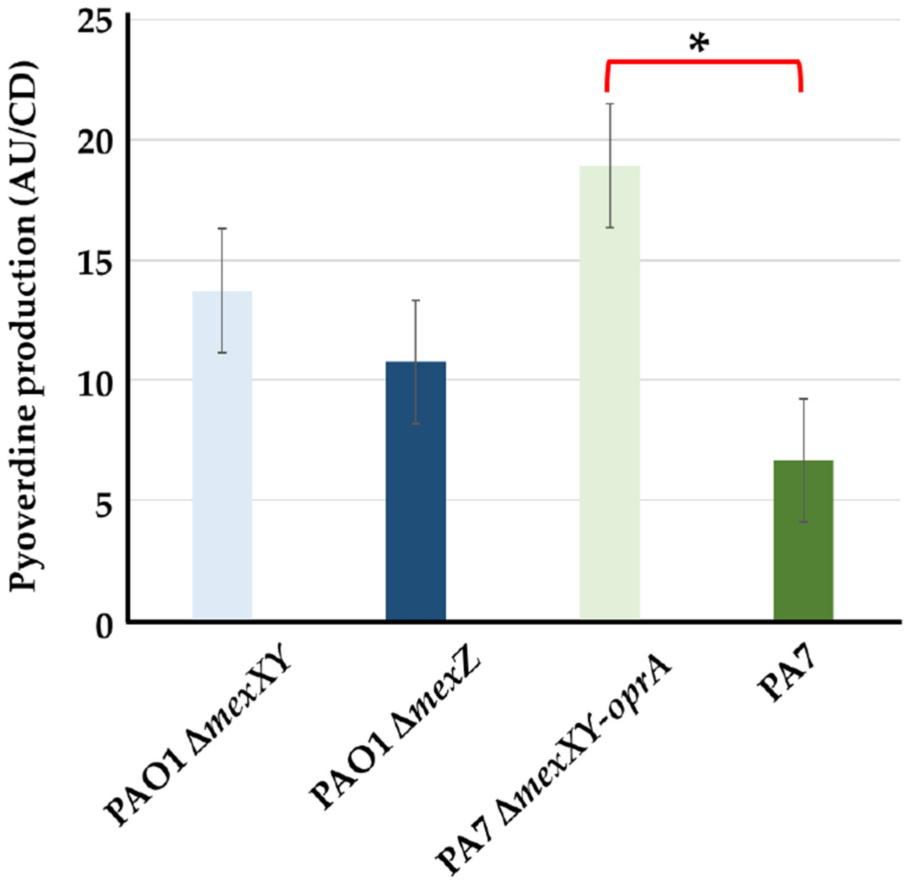

Figure S1. The MexXY multidrug efflux system decreased pyoverdine production from liquid culture in *P. aeruginosa*. Pyoverdine production was calculated by dividing the fluorescence value (arbitrary units, AU) by cell density (CD). The error range shown in the bar graph is the standard error (n = 3). * Indicates $p < 0.05$.
